# Dietary Restriction Ameliorates Age-Related Increase in DNA Damage, Senescence and Inflammation in Mouse Adipose Tissuey

**DOI:** 10.1007/s12603-017-0968-2

**Published:** 2017-09-02

**Authors:** A. Ishaq, J. Schröder, N. Edwards, T. von Zglinicki, Gabriele Saretzki

**Affiliations:** The Ageing Biology Centre, Newcastle Institute for Ageing, Institute for Cell and Molecular Biosciences, Campus of Ageing and Vitality, Edwardson Building, Newcastle upon Tyne, NE4 5PL UK

**Keywords:** Visceral fat, ageing, dietary restriction, senescence, inflammation

## Abstract

**Abstract:**

Ageing is associated with redistribution of fat around the body and saturation of visceral adipose depots. Likewise, the presence of excess fat in obesity or during ageing places extra stress on visceral depots, resulting in chronic inflammation and increased senescence. This process can contribute to the establishment of the metabolic syndrome and accelerated ageing. Dietary restriction (DR) is known to alleviate physiological signs of inflammation, ageing and senescence in various tissues including adipose tissue.

**Objectives:**

Our pilot study aimed to analyse senescence and inflammation parameters in mouse visceral fat tissue during ageing and by short term, late-onset dietary restriction as a nutritional intervention.

**Design, measurements:**

In this study we used visceral adipose tissue from mice between 5 and 30 months of age and analysed markers of senescence (adipocyte size, γH2A.X, p16, p21) and inflammation (e.g. IL-6, TNFα, IL-1β, macrophage infiltration) using immuno-staining, as well as qPCR for gene expression analysis. Fat tissues from 3 mice per group were analysed.

**Results:**

We found that the amount of γH2A.X foci as well as the expression of senescence and inflammation markers increased during ageing but decreased with short term DR. In contrast, the increase in amounts of single or aggregated macrophages in fat depots occurred only at higher ages. Surprisingly, we also found that adipocyte size as well as some senescence parameters decreased at very high age (30 months).

**Conclusions:**

Our results demonstrate increased senescence and inflammation during ageing in mouse visceral fat while DR was able to ameliorate several of these parameters as well as increased adipocyte size at 17.5 months of age. This highlights the health benefits of a decreased nutritional intake over a relatively short period of time at middle age.

## Introduction

In our rapidly ageing population more than 50% of the adult population is overweight or obese. A better understanding of the influence of ageing and nutritional interventions such as dietary restriction is important in order to counteract clinical symptoms of obesity and dysregulated metabolism. Obesity is a chronic disease that is associated with an increased risk of metabolic disorders such as insulin resistance and chronic inflammation. It is also a major driver of ageing and the development of age-related diseases. Chronic inflammation is also an important hallmark of the ageing process and is known as “inflammaging” (1). A decrease in body weight by nutritional means such as reduction of calories has been shown to have many beneficial effects on health and lifespan in various model organisms (for review see (2)). Likewise, the eradication of senescent cells has shown similar beneficial effects in mice (3).

White adipose tissue (WAT) forms an endocrine organ with both positive and negative effects on metabolism. It serves as a repository of free fatty acids (FFAs) as an energy supply. Storage and lipolysis of lipid droplets for β-oxidation in adipocytes are critical regulators of metabolic homeostasis. By secreting adipokines, adipocytes regulate metabolism, energy intake, and fat storage (4). Adipocytes are known to enlarge during obesity and the ageing process (5, 6). In contrast, caloric restriction results in decreased body mass, and preferentially reduced the mass of different fat depots including up to 78% in visceral fat (7-10). Several studies demonstrated that increased fat cell size is a significant predictor of altered blood lipid profiles and glucose-insulin homeostasis. The contribution of visceral adiposity to these associations seems to be of particular importance (11).

Senescence and inflammation are two important mechanisms contributing to ageing and the metabolic consequences of obesity. Inflammation can result from accumulation of macrophages in adipose tissue via production of cytokines such as TNFα and IL-6 (12, 13). Increase in lipolysis has been shown to induce macrophage migration in vitro (14). Macrophage numbers in adipose tissue also increase with obesity and ageing where they scavenge dead or senescent adipocytes and form aggregates and crown-like structures (15, 16). However, inflammatory cytokines and chemokines are also characteristics of the senescence-associated secretory phenotype (SASP) in senescent cells (13).

We have shown previously that ROS, DNA damage and mitochondrial dysfunction are instrumental to maintain cellular senescence (17) while eradicating mitochondria from fibroblasts significantly delayed the onset of the senescence phenotype and downregulated multiple SASP factors (18).

Various treatments have been suggested to delay senescence in adipose tissues while obesity and short telomeres exacerbated senescence (19) A recent study showed that feeding a high-fat diet ad libitum induced senescence in mouse visceral adipose tissue which could be ameliorated by exercise (20). However, DR seems to regulate many more genes than exercise in subcutaneous fat in humans (21).

We have demonstrated previously that short-term dietary restriction in wild type mice decreased the amount of senescent cells in various tissues (22). We hypothesise that pro-inflammatory cytokines and senescence are also causally related in visceral WAT, increase together during ageing, and might be rescued during DR. We used visceral WAT from mice of different ages as well as mice on late-onset, short term DR (22) to investigate the changes in adipocyte size, accumulation of γH2A.X DNA damage foci during ageing and DR, together with the expression of pro-inflammatory cytokines TNFα, IL-6, IL-1β, and senescence markers p16 and p21. We also analysed AMPK activity which is an important signal transduction pathway implicated in the regulation of physiological processes of DR (23). AMPK activation is thought to be able to inhibit inflammatory responses (24)and plays a central role in the regulation of whole body energy homeostasis and functions as a key regulator of intracellular fatty acid metabolism (25-28).

## Materials and Methods

Unless otherwise stated, all reagents were obtained from Sigma (Sigma-Aldrich, UK).

### Mice and treatments

Male mice used were of the C57Bl/6 (ICRFa) genotype (22, 29), an inbred strain previously kept as an ageing mouse colony at the Campus for Ageing and Vitality. They were housed in 56cm x 38cm x 18cm cages (North Kent Plastics, UK), each holding 4-6 mice. All were provided with paper bedding, sawdust and water, with temperature at 20°C and a 12 hour light/dark photoperiod as described in Cameron et al. (30). Standard rodent chow pellets (Special Diets Services, UK) were provided to ad libitum (AL) fed mice as described in the above paper, while DR consisted of a 26% reduced intake for 2.5 months starting at 15 months of age (22). The project was approved by the Faculty of Medical Sciences Ethical Review Committee, Newcastle University. It was licenced by the UK Home Office (PPL 60/3864) and complied with the principles for the care and use of laboratory animals.

### Tissue processing

Visceral fat tissue was obtained from mice corresponding to 5 age groups – 5 months, 17.5 months, 17.5 months DR, 24.7 months and 30 months (for histology and immunofluorescence) or 33.5 months (frozen tissue samples), respectively. Visceral abdominal fat was removed during dissection, either snap frozen or fixed in 4% paraformaldehyde (PFA) and embedded in paraffin. For all analysis methods, at least 3 mice per group were used.

### Histology and immunohistochemistry

Paraffin sections were cut at 5 μm thickness. Slides were dewaxed and hydrated in Histoclear, 100% methanol, 90%, 70%, and dH_2_O for 5 minutes each, twice. The slides were then microwaved in citrate buffer for 4 minutes on high power, and 10 minutes at 40% power.

### Haematoxylin and Eosin staining

A standard haematoxylin (Mayer’s) and eosin staining technique was used (31). After drying, slides were mounted with DPX mounting agent (Leica Biosystems, Germany) and visualised using a Nikon Eclipse E800 microscope (Nikon, Japan) at 200x magnification. 7-14 images were taken of different areas on each slide.

### CD68/Nova Red staining, imaging and analysis

Following antigen retrieval, the samples were washed with PBS and placed in 0.9% H_2_O_2_ for 30 minutes. The samples were washed and blocked for 30 minutes (5% NGS, 1% BSA in PBS). The samples were then rinsed with PBS and blocked with an avidin/biotin solution (SP-2001, Vector Laboratories, USA). The samples were then incubated overnight at 4°C in 1:100 of CD68 primary antibody (ab125212, Abcam, UK) in blocking solution (5% NGS, 1% BSA in PBS). The samples were then washed three times and incubated for 1 hour at room temperature in 1:200 of biotinylated anti-rabbit antibody (Vectastain ABC kit, Vector laboratories, USA) in blocking solution. The AB complex (Vectastain ABC kit) was then applied to the samples for 30 minutes. The samples were then washed three times. Nova Red (Vector Laboratories, USA) was then applied to the samples for exactly 2 minutes. Nova Red was rinsed off with water and methyl green (0.2% in dH2O, Sigma-Aldrich, UK) was applied to the samples for 10 minutes. The samples were then briefly rinsed with water and dehydrated in 70%, 95%, 100% methanol and Histo-Clear for 30 seconds at each step. The samples were mounted in DPX (Leica Biosystems, Germany) and stored at room temperature.

Bright field imaging was performed for the Nova Red staining using a Nikon E800 wide-field microscope (Nikon, Japan) at 200x magnification. Images were analysed using ImageJ and the cell counter plugin. The areas and perimeter of whole cells stained by H&E were traced and measured using ImageJ.

Macrophages, aggregates, and crown-like structures (CLS) were counted using ImageJ. CLS were considered as aggregates as well. CLS always contain fat molecules in the middle surrounded by macrophages due to the digestion of adipocytes.

### Immunofluorescence analysis

Slides were dewaxed and hydrated in Histoclear, 100% methanol, 90%, 70%, and dH2O for 5 minutes each, twice. The slides were then microwaved in citrate buffer for 4 minutes on high power, and 10 minutes at 40% power.

The slides were then washed in PBS for 15 minutes thrice and incubated in blocking solution (5% NGS, 1% BSA in PBS) for 1 hour. The sections were then incubated with 100 μl rabbit primary γH2A.X antibody (Cell Signalling Technologies, USA) at 1:250 in PBS mix with the same concentrations of NGS and BSA as above overnight at 4˚C. The samples were then washed with PBS for 15 minutes thrice and incubated with 100 μl goat anti-rabbit secondary antibody (AlexaFluor 488; Molecular Probes, UK) at 1:1000 5% NGS, 1% BSA PBS mix for 1 hour. The sections were then washed with PBS for 15 minutes thrice and incubated with 100 μl of DAPI (Partec, Germany). After a 10 minute wash, the sections were mounted with Vectashield (Vector Labs, USA). The slides were imaged using a wide-field fluorescence microscopy (Leica DMi8, Leica Microsystems, Germany) at 630x and 1000x magnification. All images were obtained as z-stacks with 0.45 μm thickness to encompass the entire depth of the cell, which took between 15 and 30 steps. The images were then processed using ImageJ. For γH2A.X, the blurred images at either extremes of the z-focus were removed. Remaining images were compressed onto one plane and the total foci in each nucleus was counted across 7 z-stacks (fields of capture). Each nucleus was also marked as positive or negative for foci. Percentage nucleus positive for foci, and average foci per nucleus were then determined.

**Table 1:**
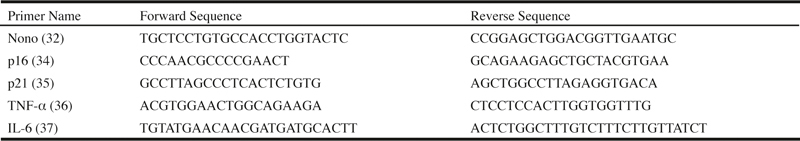
Primer sequences used in qPCR

### RNA extraction and Reverse Transcription

Up to 100 mg of whole frozen tissue samples were powderised by mortar and pestle in liquid nitrogen before RNA extraction using the Qiagen RNeasy Lipid Tissue Mini Kit (Qiagen, Belgium). The concentration of RNA obtained from the fat tissue was quantified using a Nanodrop spectrophotometer (ND-1000, Thermo Scientific, USA). 1 μg of RNA was then added to 1 μl of Random Primers (Thermo Scientific, USA) and made up to a final volume of 11 μl in nuclease-free water. RNA was denatured for 7 minutes at 75°C in a PCR Sprint Thermal Cycler (Hybaid, Germany) and briefly cooled on ice. 4 μl of 5x First Strand Buffer (Invitrogen, UK) and 2 μl of 0.1 M DTT (Invitrogen, UK) were then added to the PCR tube, along with 1 μl 10 mM dNTP mix (Biolabs, USA), 1 μl RNase Inhibitor (Promega, USA) and 1 μl Reverse Transcriptase (Superscript III; Invitrogen, USA) to a final volume of 20 μl. The reverse transcriptase mixture was then incubated at 42 ˚C for 90 minutes in the Thermal Cycler, inactivated at 95 ˚C, and the resulting cDNA stored at -70 ˚C.

### Quantitative PCR

Each sample and negative control was measured in triplicate. For each well on a 96-well qPCR plate (Applied Biosystems, USA), 5 μl of Sybr Green (SensiFAST SYBR Hi-ROX kit, Bioline, UK), 0.5 μl each of 10 μM forward and reverse primers (Table 1) diluted 1:10 in nuclease free water, and 3 μl of nuclease free water (Qiagen, Belgium) was added to a 500 μl microfuge tube. 9 μl of this mastermix was added to each well, along with 1 μl of sample cDNA. Amplification was performed with the following program: 1 cycle at 95 ˚C for 2 min, 50 cycles at 95 ˚C for 5 s and 60 ˚C for 30 s, 1 cycle at 95 ˚C for 15 s, 60 ˚C for 1 min, and 95 ˚C for 15 s. Non-POU domain-containing octamer binding protein, encoded by Nono, was used as the internal fat-specific housekeeping gene [32]. Values were expressed as a 2-ΔΔCt average for each sample, as previously described (33). Mean and SEM were then calculated for each group of mice.

### Western blotting

Protein concentrations of samples lysed in CHAPS buffer were determined using Bradford reagent (BioRAD, USA).

The Western blot protocol used was described previously (38). A 10% resolving gel was used. 50 μg of protein were loaded per well. Primary antibodies against total AMPK and phosphorylated AMPK (1:750 phosphorylated AMPK and total AMPK, Abcam, UK) were used. Western blot images were acquired using the LAS4000 imager (GE Healthcare Life Sciences, USA). Total AMPK and phosphorylated AMPK were acquired at 20 seconds. The intensity and background of each band was then measured using the AIDA software (Raytest). Phosphorylated AMPK intensities were then divided against total AMPK to obtain the ratio of phosphorylated AMPK vs total AMPK.

### Statistical Analysis

Statistical analysis was performed using SigmaPlot 12.5 (Systat Software Inc, USA). All data sets were tested for normal distribution using Mann-Whitney rank sum test, then analysed with One Way ANOVA, and either Dunn’s, Holm- Sidak, or Tukey’s tests post-hoc tests for pairwise comparisons.

The mean of normally distributed data is presented in bar charts with standard error (n=3), whereas data lacking normal distribution is presented as individual entries in box and whisker plots

## Results

### Changes in visceral adipocyte size

**Figure 1:**
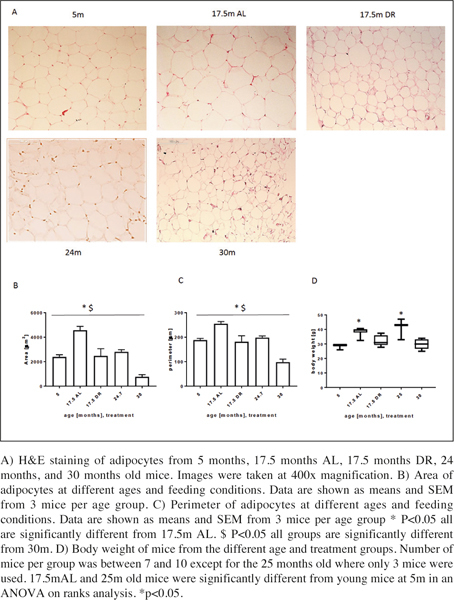
Differences in area and perimeter in adipocytes and body weights from young, old and DR mice

Histological analysis showed an increase in adipocyte size during ageing from 5 months old mice until 17.5 months (see Figure 1A for representative images). As expected, adipocyte area (Figure 1B) and perimeter (Figure 1C) correlated very well with each other (R^2^=0.967). In contrast, at higher age (around 25 months) adipocyte size got smaller and was comparable to the young group (Figure 1B, C). Surprisingly, adipocytes at very old age around 30 months were even smaller than in young mice. Thus, the adipocyte size did not just follow the pattern of body weights for the respective age groups (Figure 1D). Short term DR resulted in a significant decrease in adipocyte size at 17.5 months and was comparable to adipocytes from 5 month old mice corresponding to their reduced body weight which was also similar to that of young mice at 5 months of age.

### DNA damage and expression of senescence markers in visceral WAT

**Figure 2:**
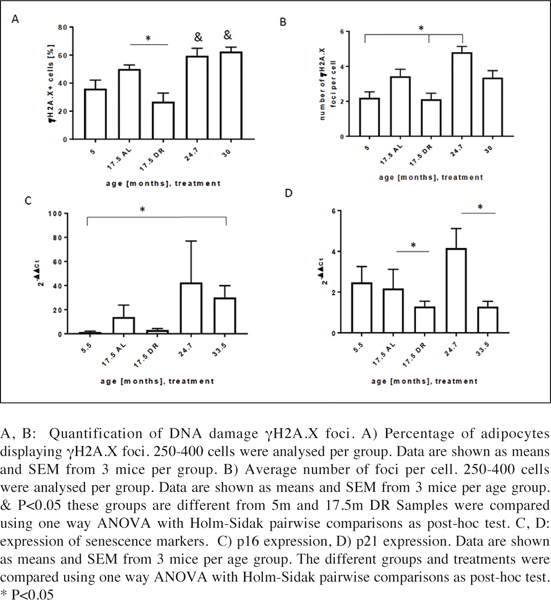
DNA damage and expression of senescence markers in adipocytes from young, old and DR mice

We have previously shown that γH2A.X DNA damage foci can be used as marker to characterise senescence in tissues (22, 39). Thus, we quantified changes in the percentage of cells harbouring DNA damage foci (Figure 2A) as well as the average number of foci per nucleus during ageing and DR in visceral WAT (Figure 2B). As expected from other tissues such as liver and gut (22) the percentage of γH2A.X positive cell nuclei increased with age up to 25 months with no further increase at 33 months of age (Figure 2A). In contrast, the 17.5 month DR visceral WAT showed a significantly reduced amount of γH2A.X-positive cells compared to 17.5 month AL.

The average number of foci per cell in general correlated well with age between 5 months and 25 months (Figure 2B). However, at 30 months of age the foci number decreased to levels similar to those in adipose tissue from 17.5 months old mice under AL.

A similar pattern was seen for expression changes of the senescence marker p16 and p21; they tended to increase with age up to 25 months but to decrease in the fat tissues from the very old (33 months) animals, and they tended to be lower following DR, although the changes were not always significant (Figure 2 C, D).

### Expression of inflammation markers

**Figure 3:**
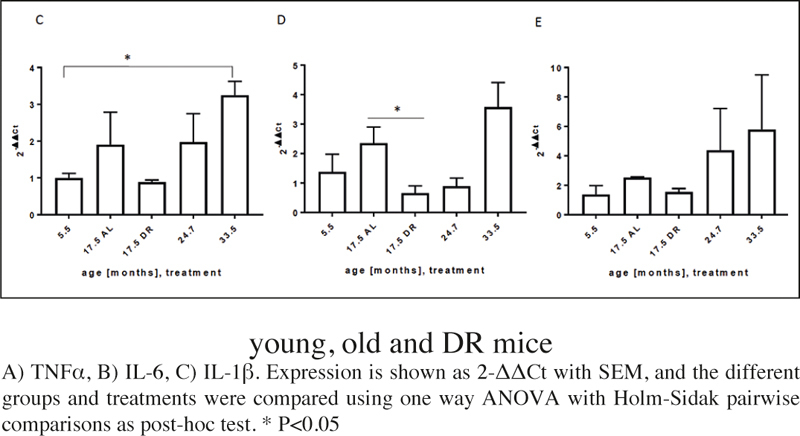
Expression of inflammation markers in visceral WAT from young, old and DR mice

Inflammation is an important hallmark of aged adipose tissue (40). Thus, we analysed the expression levels of various inflammation markers in visceral fat tissue from mice at different ages and under short term, late onset DR. All markers tended to increase with age and to decrease under DR. In contrast to the senescence markers (Figure 2), there was no tendency for inflammation to decrease in very old age (Figure 3A-C).

### Macrophage infiltration into visceral fat

In order to complement our expression analysis of inflammation markers in adipose tissue we also analysed macrophage infiltration into WAT. Infiltrating macrophages are known to be responsible for WAT inflammation, in particular the production of IL-6 (41).

**Figure 4:**
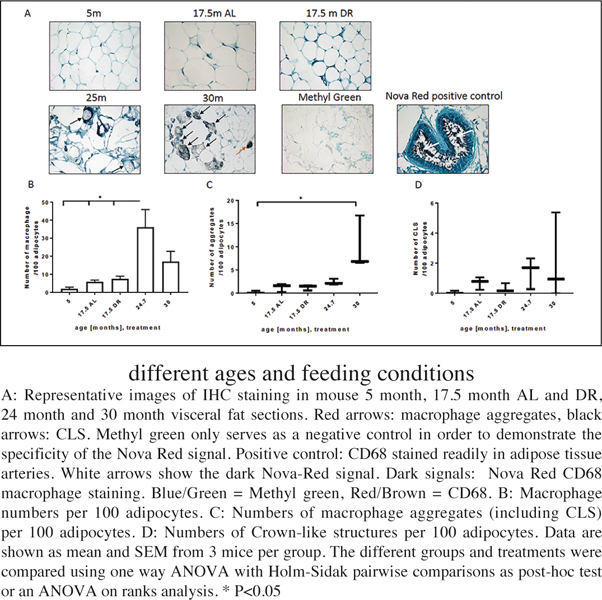
Analysis of CD68 macrophage infiltration into adipose tissue of different ages and feeding conditions

Macrophage infiltration into WAT was detected using an antibody against the macrophage transmembrane marker CD68. Representative images including a negative control without the antibody showing only the histological stain with methyl green as well as a positive control of an artery filled with macrophages are shown in fig. 4A. There were no significant differences in macrophage infiltration, aggregate number or CLS formation at younger ages or due to DR. However, we found a significantly increased amount of single macrophages at 24.7 months which tended to decrease again after that to lower levels at 30 months (Figure 4B). Probably, this late decrease was due to an increased formation of aggregates (Figure 4C) and, possibly, crown like structures (Figure 4D) at 30 months of age.

### AMPK activation

AMPK activity is regulated via AMPK phosphorylation by decreased energy (ATP) and an increased AMP level. In addition, in adipocytes AMPK can be activated by adipocytederived hormones such as leptin and adiponectin (42).

**Figure 5:**
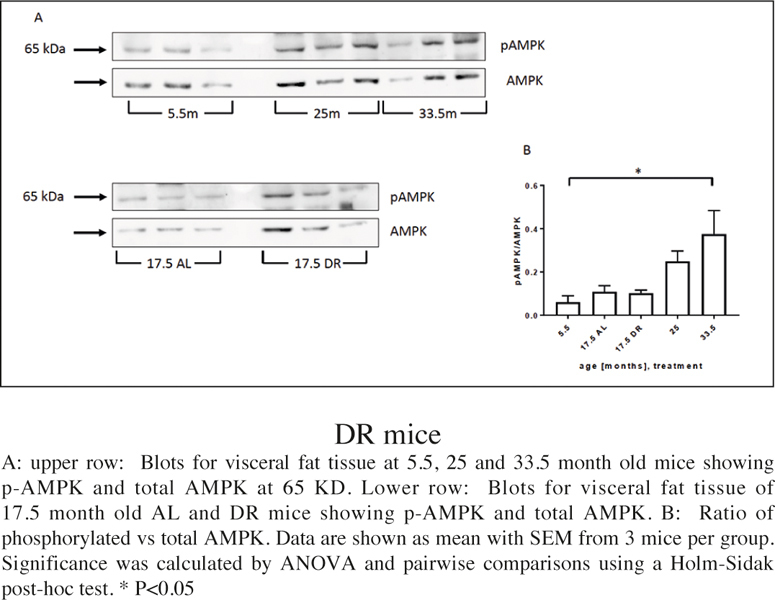
AMPK phosphorylation in visceral WAT from young, old and DR mice

We found a general trend for AMPK phosphorylation/ activity to increase during ageing reaching significance at the highest age (Figure 5B). There was no significant increase yet at 17.5 months and no change under DR.

## Discussion

As adipocytes enlarge during obesity and ageing (6, 11) adipose tissues undergo molecular and cellular alterations affecting systemic metabolism. Small adipocytes in lean individuals promote metabolic homeostasis while the enlarged adipocytes of obese and aged individuals recruit macrophages and promote inflammation and the release of various factors predisposing toward insulin resistance (43).

Changes in adipocyte size occur during ageing, and our data confirms this finding for middle-aged mice at 17.5 months while it decreased to comparable sizes as in young mice in higher age groups. This finding corresponds to that from others (6) who found a decrease of adipocyte size in subcutaneous and gonadal fat from C57BL6 mice correlating to lower body weight at 24 months. Our mouse strain is particularly long lived (44) and thus their body weight decreases only at a higher age.

Others have found that telomere lengths in adipocytes was inversely correlated to adipocyte size and waist circumference in obese subjects (45). However, our results suggest that adipocyte size per se is not a good marker for measuring agerelated changes in WAT of mice at higher ages. A reason for this could be a re-distribution of fat in the body during ageing due to increased triacylglyceride accumulation in the liver and less in subcutaneous fat (46). Liver steatosis due to ageing and cellular senescence can be greatly prevented and ameliorated with dietary restriction and pharmacological eradication of senescent cells in the liver (47).

DR was able to significantly reduce adipocyte size and multiple marker of adipocyte senescence (significant for DNA damage, p21 and IL-6 expression). This indicates that DR acts as a senolytic treatment in visceral fat, similar to its effects in other tissues like liver, intestine or corneal epithelium (22, 47, 48). There was no change in frequencies of single or aggregated macrophages under DR and only a tendency towards a decrease in CLS from a rather low level, suggesting that DR reduces frequencies of senescent adipocytes not by activating their immuno-surveillance, at least not macrophagemediated immuno-surveillance. This is in contrast to the transient increase in macrophage infiltration due to increased lipolysis during weight loss and fasting of obese mice found by others in perigonadal WAT (49). However, it is known that different fat depots can react differently to dietary interventions such as DR (50) and that both senescent cell frequencies (5) and IL-6 secretion (51) are higher in visceral than in peripheral or subcutaneous fat depots. Moreover, the effects of DR on adipocyte senescence and pro-inflammatory cytokines were not associated with different activation of AMPK. This result is in contrast to data from others who described an increase in AMPK activation after short term DR (5 weeks) in genetically obese mice (52). Likewise, other have found a decrease of activated AMPK in epididymal WAT from obese mice and wild type mice fed a high fat/high sucrose diet for 4 months (53).

In hepatocytes and fibroblasts, DR may suppress induction of senescence by improving mitochondrial fatty acid turnover (47). A similar mechanism appears possible for adipocytes. Targeted organism-wide depletion of senescent cells (including WAT) normalised various tissue parameters including expression of adipogenesis markers to that of young mice (3). Together, this suggests that important metabolic benefits of DR, including improvements in glucose tolerance and insulin resistance, might be mediated by reduction of adipocyte senescence. The effect of nutritional interventions such as by feeding mice a high fat diet on the increase of adipocyte size, senescence markers (p21, p53 and p16) as well as proinflammatory SASP factors (including IL-6) in visceral fat tissue has been recently shown by Schafer et al. (20). The authors also showed a decrease of the senescent phenotype by exercise.

In contrast to our expectations and to the continuous increase of senescent cell frequencies up to very old age (42 months) in liver and intestine of mice (54, 55), we here observed decreases in several markers of adipocyte senescence at advanced age (30 – 33 months), but no decrease in the expression levels of multiple pro-inflammatory cytokines and a continuing increase in AMPK phosphorylation. To our knowledge so far AMPK activity in visceral fat from old mice has not yet been described and we were surprised to see an increase there. We speculate that this could be associated to lower adipocyte size at higher ages (see figure 1).

At the same time, there were high numbers of infiltrating macrophages, many of which were aggregated or forming CLS, which might be responsible for the continuously high levels of inflammation mediators. These macrophages could also be responsible for the removal of cells with very high amounts of DNA damage foci (see fig. 2B). This data might indicate that macrophages in visceral fat need a relatively high threshold of signals from senescent adipocytes to become activated, and that this threshold is only reached at very advanced age. Alternatively, increased AMPK activation at old ages might drive fatty oxidation, lipolysis and redistribution of fatty acids to liver (6, 46, 47, 56). A more comprehensive analysis of SASP and macrophage-derived inflammation markers would be required to draw a definitive conclusion.

*Acknowledgements:* The work was supported by BBSRC grant BB/C008200/1 to TvZ and from a Grant from Newcastle University Institute for Ageing (BH161774) to GS

*Disclosure statement:* None of the authors have anything to disclose

*Ethical standard:* All experiments complied with current laws of the United Kingdom.
